# LC-NMR Technique in the Analysis of Phytosterols in Natural Extracts

**DOI:** 10.1155/2013/526818

**Published:** 2013-12-23

**Authors:** Štěpán Horník, Marie Sajfrtová, Jindřich Karban, Jan Sýkora, Anna Březinová, Zdeněk Wimmer

**Affiliations:** ^1^Institute of Chemical Process Fundamentals of the ASCR, v.v.i., Rozvojová 2/135, 16502 Prague 6, Czech Republic; ^2^Institute of Organic Chemistry and Biochemistry of the ASCR, v.v.i., Flemingovo Náměstí 2, 16610 Prague 6, Czech Republic; ^3^Institute of Experimental Botany of the ASCR, v.v.i., Isotope Laboratory, Vídeňská 1083, 14220 Prague 4, Czech Republic; ^4^Institute of Chemical Technology Prague, Faculty of Food and Biochemical Technology, Department of Chemistry of Natural Compounds, Technická 5, 16628 Prague 6, Czech Republic

## Abstract

The ability of LC-NMR to detect simultaneously free and conjugated phytosterols in natural extracts was tested. The advantages and disadvantages of a gradient HPLC-NMR method were compared to the fast composition screening using SEC-NMR method. Fractions of free and conjugated phytosterols were isolated and analyzed by isocratic HPLC-NMR methods. The results of qualitative and quantitative analyses were in a good agreement with the literature data.

## 1. Introduction

Phytosterols are compounds naturally occurring in plants. They are structural analogues of cholesterol which is predominant in animals (although cholesterol has been found in very small amounts in plants as well). Phytosterols occur either in a free form or in the form of the so-called conjugates. The conjugated form is composed of a sterol having at position C-3 either a fatty acid (esters), or a hexose (glycosides), or a hexose with a fatty acid bonded to 6-OH of the hexose skeleton (acylated glycosides) [1, 2]. Regarding human nutrition, phytosterols are most abundant in vegetable oils and margarines, followed by vegetables, seeds, or pods [1]. They have become a subject of interest due to their biological properties. They are able to lower the cholesterol level in blood, especially low-density lipoprotein (LDL) cholesterol, thereby reducing the risk of cardiovascular diseases [3]. Phytosterols also have been proven to have antioxidant [4], anti-inflammatory [5], and antitumor effects [6, 7].

All the applications of phytosterols as dietary supplements are preceded by sophisticated analytical procedures which usually begin with the analysis of crude plant extracts. Gas and/or liquid chromatography techniques with various detectors play the pivotal role in the analysis of phytosterols. Currently, GC-FID technique prevails [8–12], followed by GC-MS [8, 9, 13, 14]. Liquid chromatography mostly uses coupled mass spectrometers [14–17] as an alternative to UV detection [18]. A comprehensive review of analytical and detection techniques utilized in the phytosterol analysis of dietary products was published by Abidi [19] and later extended by Lagarda et al. [20]. A particular analysis is usually preceded by saponification of oil or extract [16, 17, 21]. Saponification provides a concentrated sterol fraction which facilitates analysis. On the other hand, the information regarding conjugated sterols is lost as they are converted into their free form. Therefore, the usual result is the information on overall sterol composition. To the best of our knowledge, any method for simultaneous evaluation of free and conjugated sterol contents has not yet been published despite extensive research being conducted on biological properties of conjugated phytosterols [22].

Generally, GC and HPLC techniques require compound verification by an authentic sample, which might be difficult to obtain in the case of conjugated sterols. The lack of authentic samples can be compensated for by means of structure-sensitive detection techniques, for example, ^1^H NMR [23]. Most of the free phytosterols have similar signal fingerprints in ^1^H NMR spectra [24]. The same fingerprint is also preserved in the spectra of their conjugated forms but these spectra show additional signals due to substituents at position C-3 [25]. Besides giving structural/qualitative information, ^1^H NMR detection is also a quantitative method [22]. Therefore, HPLC-NMR hyphenation can also provide information about quantitative composition of a given sample.

For the purpose of simultaneous analysis of free and conjugated sterols in natural extracts, we have extended previously published LC-NMR method originally developed for the analysis of free fatty acids in natural oils [26]. We have also developed fast composition screening method using SEC-NMR technique. The advantages and disadvantages of both methods are discussed in this paper.

## 2. Results and Discussion

Generally, LC-NMR is limited by the availability of solvents in “LC-NMR” purity grade [27]. Combination of D_2_O-acetonitrile and acetonitrile-CDCl_3_ is usually used in reverse phase chromatography for the analysis of polar or nonpolar samples, respectively. The latter combination was also utilized in our method for the analysis of nonpolar phytosterols and their fatty acid conjugates. The samples were obtained by supercritical carbon dioxide extraction and therefore consisted mostly of nonpolar compounds. A mild gradient of CDCl_3_ in acetonitrile was applied to achieve sufficient separation of individual components. The separation started at 10% and ended at 90% of CDCl_3_ in 100 minutes. Other chromatographic parameters were adjusted to the requirements of the quantitative LC-NMR analysis [26], for example, flow rate 0.5 mL/min (for details see the Supplementary Material available online at http://dx.doi.org/10.1155/2013/526818).

**Figure 1:**
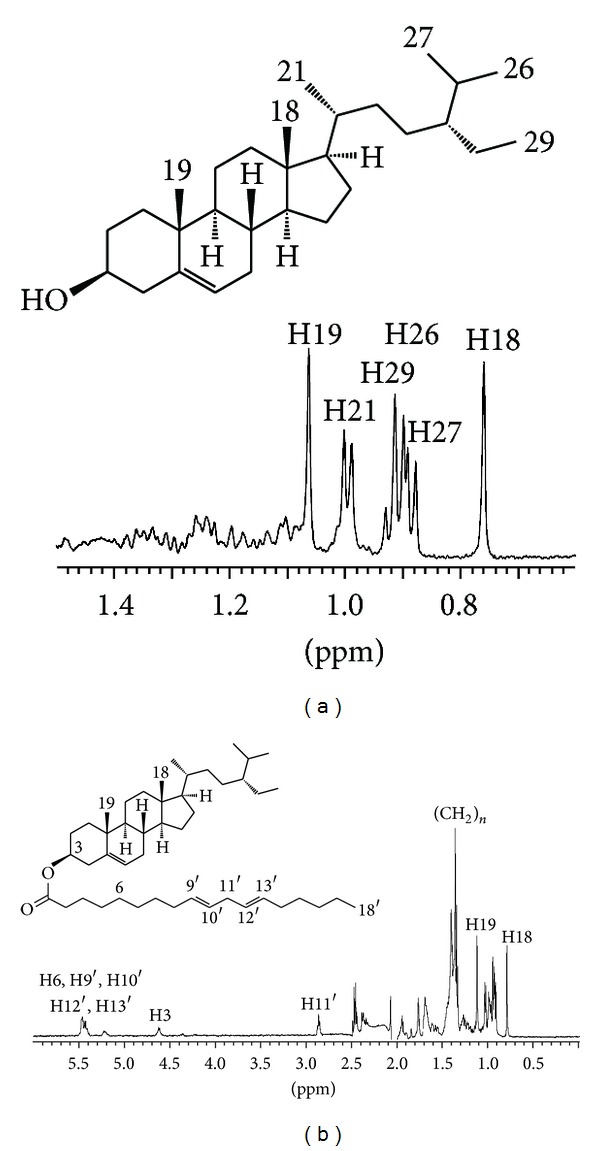
^1^H NMR spectra of free (a) and conjugated (b) *β*-sitosterols. Spectra were collected in a stop-flow LC-NMR experiment.

The stinging nettle (*Urtica dioica*) has many therapeutic effects [28] and it is also known for its relatively high content of phytosterols (namely, *β*-sitosterol) [29]. The root extract of the stinging nettle was chosen as a testing sample for our chromatographic method. Under given chromatographic conditions the free phytosterols eluted at retention time between 30 and 40 minutes followed by conjugated phytosterols whose signals were detected around 60 minutes of the separation. The predominant free phytosterol was identified as *β*-sitosterol (confirmed by off-line GC-MS). The detected conjugated phytosterol was recognized as *β*-sitosterol linoleate. The structure was deduced from ^1^H NMR data and confirmed by off-line HR-MS ([M-Na]^+^ peak, *m/z* = 699.6047; calculated *m/z* = 699.6051; see Supplementary Material). The separation was solely monitored by on-flow ^1^H NMR detection. Because the signals of the methyl groups in phytosterols occupy specific region in the ^1^H NMR spectrum (0.6–1.1 ppm), they can be easily recognized even at low concentrations and/or in the mixtures (Figure 1).

It is noteworthy that the extract was used without any derivatization or treatment; it was just dissolved in CDCl_3_ and subjected to HPLC. The on-flow arrangement of the NMR experiment provided one spectrum every 5 seconds; one spectrum is the result of accumulation of four scans. The quantitative analysis can be performed just by simple integration of a given signal across all NMR spectra. In our particular case the most upfield signal (usually H18) was chosen for integration. Estimated integration revealed that the free: conjugated phytosterol ratio was 6 : 1 in the stinging nettle extract sample. To estimate overall phytosterol content a precise calibration had to be performed.

**Figure 2:**
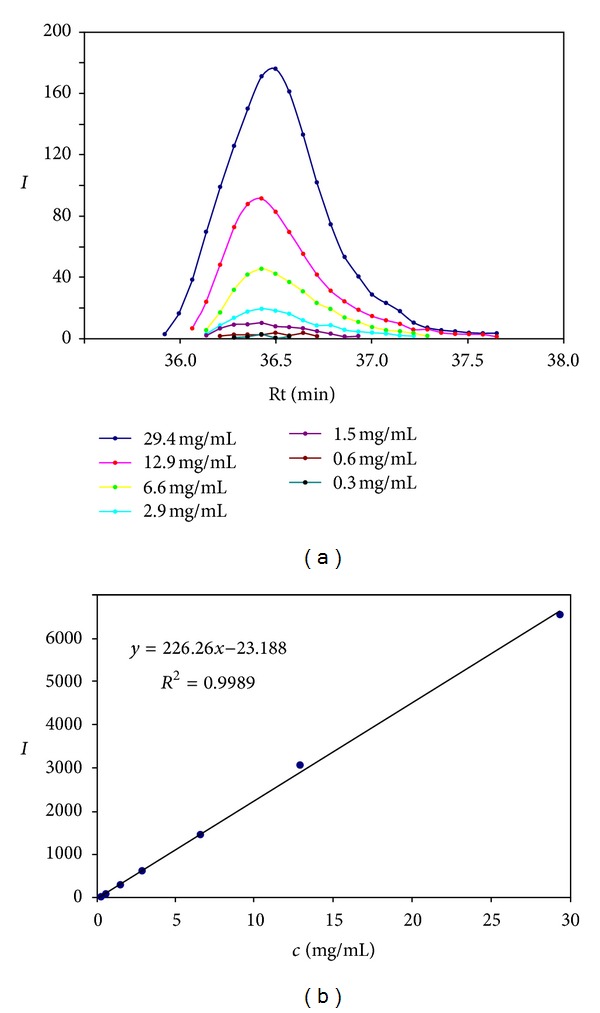
Calibration of the on-flow HPLC-NMR experiment. “*I*” on the *y*-axis stands for integral in the individual ^1^H NMR spectra (a) or overall integral obtained by numerical integration (b).

The calibration was performed with a CDCl_3_ solution of *β*-sitosterol standard (~97%, SigmaAldrich). The calibration plot obtained from measurements at seven concentration levels showed a linear response of ^1^H NMR detection covering two orders of magnitude (Figure 2). The residual CHCl_3_ signal served as an internal reference.

Although the calibration was performed only with the *β*-sitosterol standard, we presumed that the calibration line can be applied also to other phytosterols. Because the integrated signal (usually H18) originates from the methyl group at the centre of the sterol molecule its nature and the chemical environment are very similar in all phytosterols. Therefore, the relaxation properties and consequently the response for quantitation remain similar even in other types of phytosterol molecules, for example in conjugated phytosterols. The quantification limit of the method was estimated to 0.3 mg/mL.

**Figure 3:**
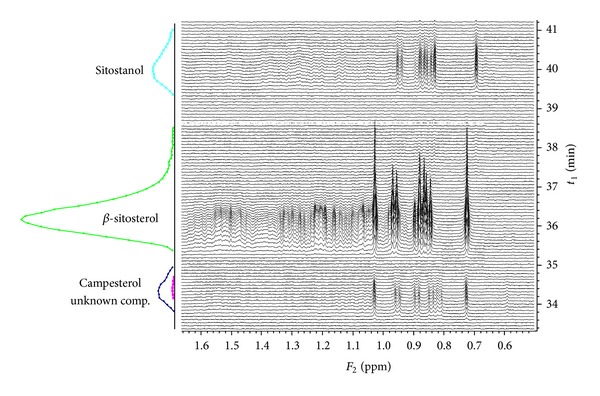
On-flow HPLC-NMR measurement of technical *β*-sitosterol and its quantitative analysis.

Technical *β*-sitosterol (~60%, SigmaAldrich) was chosen as a reference mixture as it contains two other phytosterols (campesterol and sitostanol) which can be used for further authentication of chromatographic peaks in natural samples. The separation is shown in Figure 3. The separation revealed the following composition and elution order: 6% of campesterol (34 min), 81% of *β*-sitosterol (36 min), and 12% of sitostanol (40 min). Campesterol coeluted with an unknown compound (1%) which could not be identified due to its low concentration and strong signal overlap.

**Figure 4:**
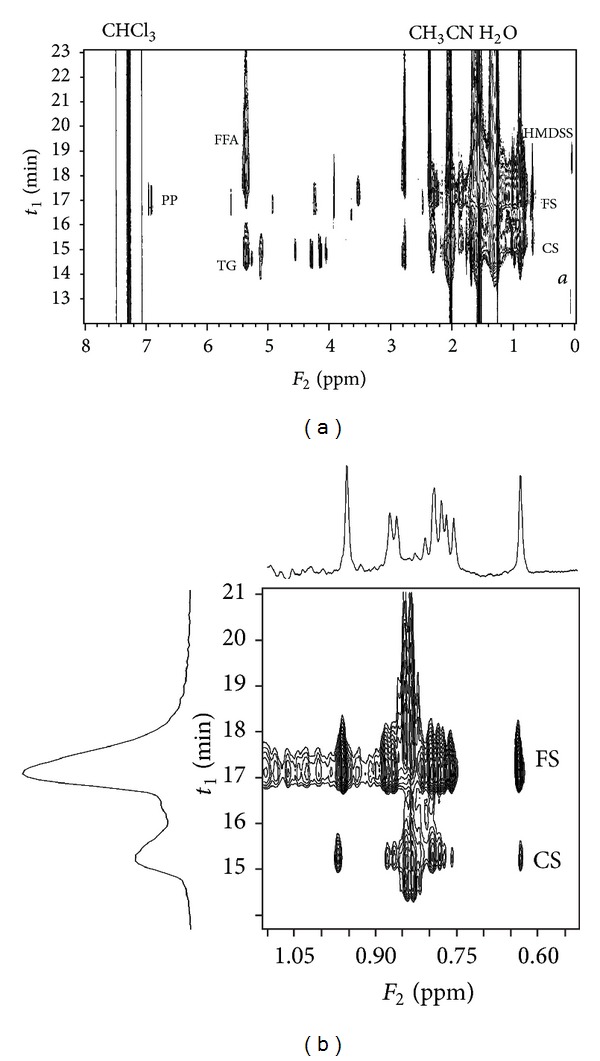
On-flow SEC-NMR measurement of the stinging nettle extract, whole spectrum (a) and a detail of the upfield region (b). FFA: free fatty acid, TG: triglycerides, PP: polyphenolic compounds, HMDSS: hexamethyldisilane, FS: free phytosterols, CS:conjugated phytosterols, and *a*: silicone grease.

The major disadvantage of the method described above is its time requirement. 90 minutes including column reconditioning is unsatisfactory time frame in the era of UPLC and/or UHPLC. Another option providing separation of free and conjugated molecules is the size exclusion chromatography (SEC). Components are separated by their different molecular size in SEC. It can also be coupled to the ^1^H NMR for structure-sensitive detection. SEC-NMR had to be run in 100% CDCl_3_ (due to the solvent purity requirements) with the flow rate 0.5 mL/min. Under these conditions the signals of conjugated phytosterols occurred at 15 minutes and the free phytosterols were detected at 17 minutes in the stinging nettle sample (Figure 4).

The SEC-NMR method seems to be reasonably fast (30 minutes) and also sensitive as we detect accumulated signals of all phytosterols present in the sample in the same region of the chemical shift. In the resulting pseudo-2D spectrum we can also recognize signals of other present molecules such as free fatty acids, triglycerides, and some polyphenolic compounds. On the other hand, we cannot identify individual components within each group of compounds due to a strong signal overlap. The calibration was performed for the purpose of quantitative analysis. It showed again linear response of ^1^H NMR detection and surprisingly a slightly higher quantification limit (~1.0 mg/mL) which is caused mainly by significant tailing of the chromatographic peaks; the chromatographic peak elutes for 2 minute in SEC-NMR (see Figure S1 in Supplementary Material) compared to 1 minute in HPLC-NMR method (Figure 2).

**Figure 5:**
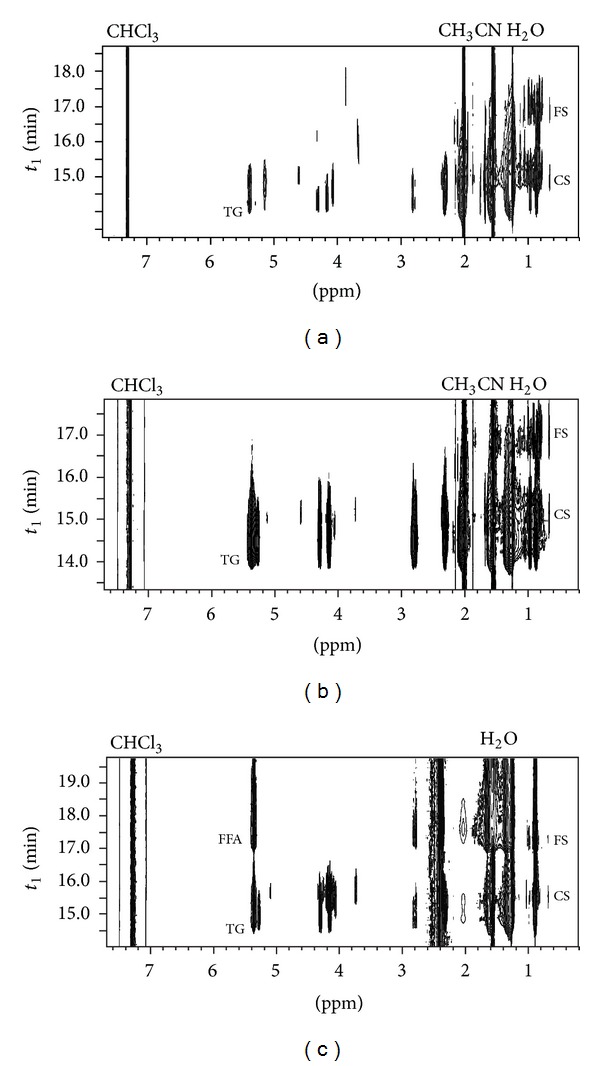
SEC-NMR measurement of the sea buckthorn extract samples, leaves (a), seed oil (b), and seed coat (c). FFA: free fatty acid, TG: triglycerides, FS: free phytosterols, and CS: conjugated phytosterols.

However, the SEC-NMR method seems to be a suitable method for fast screening of the phytosterol content, for example, in different extracts from the same plant. Thus, samples of leaves, seed oil, and seed coat of sea buckthorn (*Hippophae rhamnoides*), whose medicinal and therapeutic potential has been recently reviewed [30], were extracted by supercritical CO_2_ and the extracts were analyzed by SEC-NMR method for their phytosterol content. The results are given in Figure 5.

**Figure 6:**
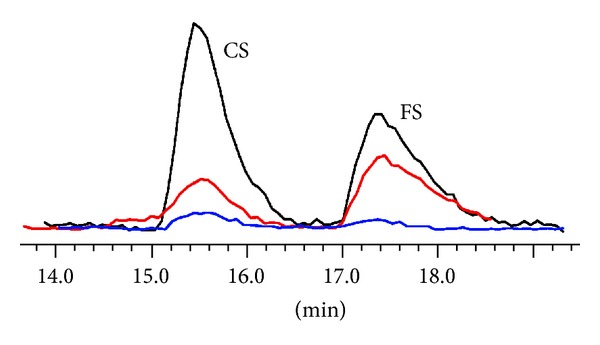
Projection of the phytosterol content in the sea buckthorn extracts (SEC-NMR measurement in 100% CDCl_3_), leaves (black), seed oil (red), and seed coat (blue). CS: conjugated phytosterols and FS: free phytosterols.

The leave extract showed the highest content of conjugated phytosterols of the three extracts; it was even higher than the content of free phytosterol in this sample. The seed oil extract contained more free phytosterols than conjugated ones. Triglycerides were the predominant compounds in this sample as expected. The seed coat extract contained triglycerides and fatty acids in large amounts and only traces of phytosterols, mostly in a conjugated form (Figure 6).

Additionally, the results of SEC-NMR analyses facilitate choice of an appropriate method for detailed qualitative analysis. Both groups of phytosterols can be easily isolated from the leave extract by means of preparative SEC. There is no significant coelution with other compounds in this sample. The seed oil extract is also rich in phytosterol; however, phytosterol isolation by means of SEC would be impractical due to the high content of triglycerides which would prevail in the fraction of conjugated phytosterols. The seed oil extract was therefore saponified [31] and analyzed for its overall phytosterol composition. The seed coat extract was excluded from further investigation as its phytosterol content was negligible.

Saponified seed oil extract was dissolved in CDCl_3_ and subjected to HPLC-NMR. The isocratic conditions provided sufficient separation of phytosterol content (CDCl_3_ : acetonitrile, 25 : 75). Nine major compounds were found, seven of them were fully identified, one was assigned to a compound family, and one compound remained unidentified. The assignment was based mainly on ^1^H NMR spectral patterns and was confirmed by an off-line GC-MS measurement. ^1^H NMR spectra of identified phytosterols are shown in Figure S2 in Supplementary Material. *β*-Sitosterol was identified as a main phytosterol in the seed oil. The found composition, listed in Table 1, is in good agreement with the published data [32].

The fraction of free phytosterols isolated by preparative SEC from the leave extract showed completely different composition. Signals of seven phytosterols were observed, three polar phytosterols (erythrodiol, uvaol, and oleanolic aldehyde), one unidentified, and three common phytosterols (*α*- and *β*-amyrin and *β*-sitosterol). ^1^H NMR spectra of identified phytosterols are shown in Figure S2 in Supplementary Material. The most abundant phytosterol in this fraction was *β*-amyrin. The comparative composition is listed in Table 2.

**Figure 7:**
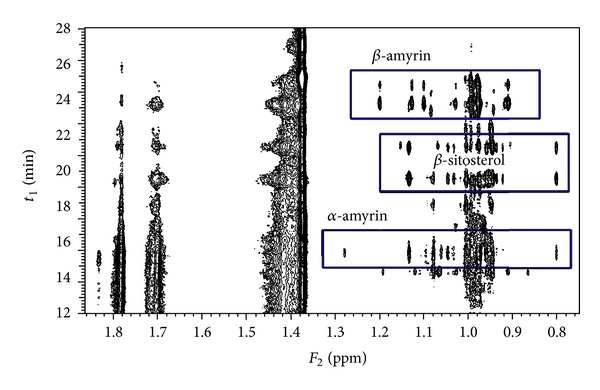
HPLC-NMR measurement of the conjugated phytosterol fraction extracted from the sea buckthorn leaves; isocratic method (CDCl_3_ : acetonitrile, 50 : 50).

The fraction of conjugated phytosterols isolated by preparative SEC from the leave extract was analyzed under different isocratic conditions (CDCl_3_: acetonitrile, 50 : 50). Five predominant conjugates were identified in the pseudo-2D spectrum (Figure 7). According to the elution order, the first compound can be attributed to conjugated *α*-amyrin, the second and third to conjugates of *β*-sitosterol, and the last two to conjugates of *β*-amyrin. The comparative composition is listed in Table 3. It is apparent that the conjugate with unsaturated fatty acid elutes before that with saturated fatty acid. According to the integration of ^1^H NMR signals these fatty acids are probably linoleic and palmitic acids. This has to be confirmed by HR-MS. The overall composition of isolated conjugates is in good correlation with the composition found in free phytosterols confirming *β*-amyrin as the most populated phytosterol in the sea buckthorn leaves.

## 3. Conclusion

^1^H NMR spectroscopy was shown to be a suitable detection technique in the analysis of various phytosterol forms in natural extracts. The HPLC-NMR method can be utilized in the qualitative analysis of phytosterols when the structural information is necessary, whereas the SEC-NMR method can be used for the fast composition screening. The main disadvantage of ^1^H NMR as a detection technique is its low sensitivity.

## Supplementary Material

Electronic Supporting Information—ESI
The ESI contains list of used chemicals and all experimental details of LC-NMR apparatus and method, GC-MS, HR-MS, supercritical fluid extraction, and saponification procedure.

## Figures and Tables

**Table 1:** Composition of the saponified sea buckthorn seed oil determined by HPLC-NMR.

Compound	Comparative content^a^	Overall content in the seed oil^b^	Retention time (min)^c^
Δ^5^-avenasterol	13%	0.3%	24
Unknown I	2%	<0.1%	25
Unknown II (Δ^7^-sterol)	1%	<0.1%	25
Cykloeukalenol	4%	0.1%	26
*α*-amyrin	4%	0.1%	27
Campesterol	2%	<0.1%	27
*β*-amyrin	3%	<0.1%	28
*β*-sitosterol	69%	1.7%	29
Sitostanol	2%	<0.1%	32

^a^Molar ratio in the phytosterol fraction, ^b^weight ratio in the extract sample, and ^c^isocratic method (CDCl_3_ : acetonitrile, 25 : 75).

**Table 2:** Composition of the fraction of free phytosterols in the sea buckthorn leaves determined by HPLC-NMR.

Compound	Comparative content^a^	Overall content in the leave extract^b^	Retention time (min)^c^
Erythrodiol	3%	<0.1%	13
Uvaol	14%	0.3%	14
Oleanolic aldehyde	4%	<0.1%	16
Unknown	3%	<0.1%	24
*α*-amyrin	12%	0.3%	27
*β*-amyrin	47%	1.0%	28
*β*-sitosterol	17%	0.4%	29

^a^Molar ratio in the phytosterol fraction, ^b^weight ratio in the extract sample, and ^c^isocratic method (CDCl_3_ : acetonitrile, 25 : 75).

**Table 3:** Composition of the fraction of conjugated phytosterols in the sea buckthorn leaves determined by HPLC-NMR.

Compound	Comparative content^a^	Overall content in the leave extract^b^	Retention time (min)^c^
*α*-amyrin + FA	11%	0.6%	15
*β*-sitosterol + unsat. FA	18%	1.0%	20
*β*-sitosterol + sat. FA	12%	0.6%	22
*β*-smyrin + unsat. FA	39%	2.1%	25
*β*-amyrin + sat. FA	20%	1.1%	26

^a^Molar ratio in the phytosterol fraction, ^b^weight ratio in the extract sample, and ^c^isocratic method (CDCl_3_ : acetonitrile, 50 : 50).
