# Supplementary material for: LC-NMR Technique in the Analysis of Phytosterols in Natural Extracts
Source: J Anal Methods Chem. 2013 Dec 23;2013:526818. doi: 10.1155/2013/526818 (PMC3884959; doi:10.1155/2013/526818)
Supplement: Supplementary file 1 — Electronic Supporting Information—ESI The ESI contains list of used chemicals and all experimental details of LC-NMR apparatus and method, GC-MS, HR-MS, supercritical fluid extraction, and saponification procedure. [file 526818.f1.pdf]

SUPPLEMENTARY MATERIAL

for

LC-NMR technique in the analysis of phytosterols in natural extracts

Štěpán Horník, Marie Sajfrtová, Jindřich Karban, Jan Sýkora, Anna Březinová and Zdeněk Wimmer

Content of Supporting Information

Used chemicals...2

Saponification...2

LC-NMR apparatus and methods

HPLC..3

SEC..3

NMR spectroscopy...4

GC-MS and HR-MS..7

Supercritical fluid extraction..8

References..8

Used chemicals

Acetonitrile, ACN, (HPLC grade for preliminary analyses and LC-NMR grade, Fluka), chloroform-d (99.5% D, water content < 0.01%, EURISO-TOP) were used as solvents for HPLC separation. β-Sitosterol (3β-stigmast-5-en-3-ol), 60% purity (residues of campesterol and β-sitostanol) was purchased from Sigma-Aldrich; 83% β-Sitosterol + campesterol and 17% of sitostanol according to the rough integration of ^{1}H NMR spectra. All reagents were used as received.

Saponification

Sea buckthorn seed oil (1 g) was mixed in 100 ml round bottom flask with 20 mL of methanolic KOH (1.0 M). The reaction mixture was stirred at ambient temperature overnight. The saponification was carried out in two batches which were subsequently combined after the reaction was complete. Combined mixture was diluted with 80 ml of water and extracted with MTBE (3 x 60 ml). Organic layer was extracted with methanolic KOH (0.5 M), water (3 x 60 ml) to neutral pH and brine (30 ml). The MTBE layer was dried with anhydrous magnesium sulfate and evaporated in a vacuum rotary evaporator at 30°C. The Procedure is based on the publication of Li et al. [S1].

LC-NMR apparatus and methods

HPLC

A commercial HPLC system (Varian ProStar 230) with two columns in series, 150×4.6 mm C_8 and 250×4.6 mm C_{18} (both Phenomenex Luna, 5 μm particles, 300 Å pore size), was employed in the HPLC-NMR experiments described. Three HPLC experiment were presented in the main article.

i) A gradient method for the simultaneous analysis of free and conjugated phytosterols. The separation started with 90:10 and changed successively to 10:90 v/v ratio (ACN:$CDCl_3$) in 100 min. All the experiments were conducted with the flow-rate of 0.5 mL/min. The calibration measurements were performed with solutions of β-sitosterol in $CDCl_3$. 8.5 mg of the β-sitosterol standard was dissolved in 0.28 mL of $CDCl_3$ (c = 30 mg/mL), and 20 μL of the solution was subjected to separation. The residual solution was diluted to approx. double volume in every successive calibration step. The final concentration measured was 0.3 mg/mL, which represented also the concentration limit for the integration. The integration of H18 methyl signal was carried out against the signal of residual solvent (CHCl3, 7.61 ppm) in each spectrum. The area of final chromatographic peak was determined by numerical integration using the rectangular rule.

ii) An isocratic method for the analysis of the fraction isolated free phytosterols. The ACN:$CDCl_3$ 75:25 was kept during whole analysis.

iii) An isocratic method for the analysis of the fraction isolated conjugated phytosterols. The ACN:$CDCl_3$ 50:50 was kept during whole analysis.

SEC

The same HPLC system (Varian ProStar 230) was used also for SEC-NMR experiments; 250 x 4,6 mm column (Polymer Laboratories, PLgel, 5 μm, 300 Å). The separation was performed in 100% $CDCl_3$ with the flow-rate of 0.5 mL/min. The calibration measurements were performed with solutions of technical β-sitosterol dissolved in $CDCl_3$.

The first solution was prepared in 12.6 mg/mL concentration. The residual solution was diluted to approx. double volume in every successive calibration step. The final concentration measured was 0.7 mg/mL. The limit for the integration was estimated to 1.0 mg/mL. The integration of H18 methyl signal (signal of campesterol and β-sitosterol together) was carried out against the signal of residual solvent (CHCl3, 7.26 ppm) in each spectrum. The area of final chromatographic peak was determined by numerical integration using the rectangular rule (Figure S1).

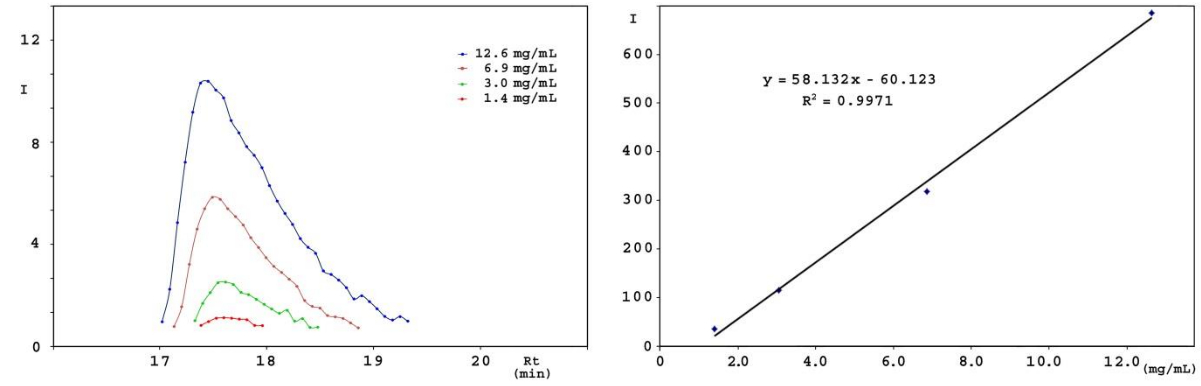

Figure S1. Calibration of the SEC-NMR method. ”I” on the Y-axis stays for integral in the individual ^{1}H NMR spectra (left) and overall integral obtained by numerical integration (right).

Real samples were prepared by dissolving the entire carbon dioxide extract in $CDCl_3$ to provide approx. 100 mg/mL concentration. For each of the separations, the injected volume was 20 μL.

^{1}H NMR detection

^{1}H NMR detection was performed on Varian INOVA 500 MHz spectrometer equipped with HCN triple resonance (60 μL active volume) microflow probe. The operating spectrometer frequency was 499.9 MHz, standard LC-NMR software vnmrC 6.1 was used throughout. The spectrometer was equipped with a X,Y,Z-Performa gradient module, had four r.f. channels, two of them fitted with waveform generators. All the separations and NMR detection were done at ambient temperature (22 ^{o}C). ^{1}H NMR data were collected in the on-flow mode employing WET multiple frequency solvent suppression [S2], to suppress signals from ACN (around δ = 2.00 ppm) and HOD (δ = 2.14 ppm). One scout scan was applied at the start of each acquisition block (usually 5–8 min). The four transients of 1 s acquisition time covering the spectral width 10 kHz that followed after 90° RF pulse (3.4 μs) were accumulated. Data were zero-filled to 32K and the initial 3 data points were obtained by backward projection (32 LP coefficients). The residual signals of water and ACN were eliminated by digital solvent subtraction before Fourier transform; sp line baseline correction was applied to the spectra. No other data processing was employed. The ^{1}H NMR signal of the residual $CHCl_3$ served as an internal reference both for chemical shifts (7.61 ppm in HPLC and 7.26 ppm in SEC experiments) and the integration, as described above. The stop-flow ^{1}H NMR data were collected with the acquisition time of 2 s and the relaxation delay of 1 s with the number of transients depending on the signal-to-noise ratio.

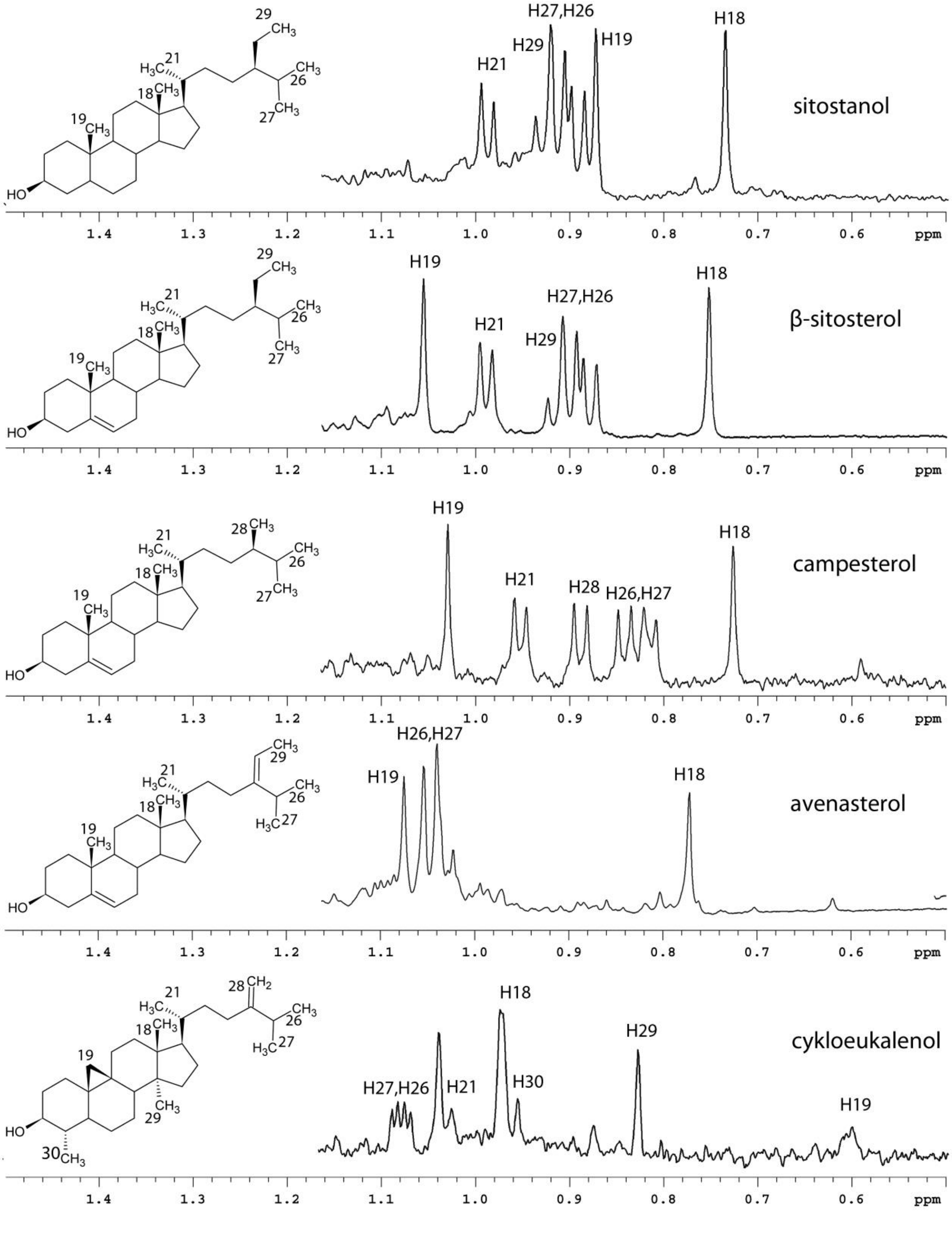
sitostanol
H27,H26
H18
H29
H19
H21
β-sitosterol
H19
H18
H27,H26
H21
H29
campesterol
H19
H18
H21
H28
H26,H27
avenasterol
H26,H27
H19
H18
cykloeukalenol
H18
H29
H30
H27,H26
H21
H19
1.4
1.3
1.2
1.1
1.0
0.9
0.8
0.7
0.6
ppm

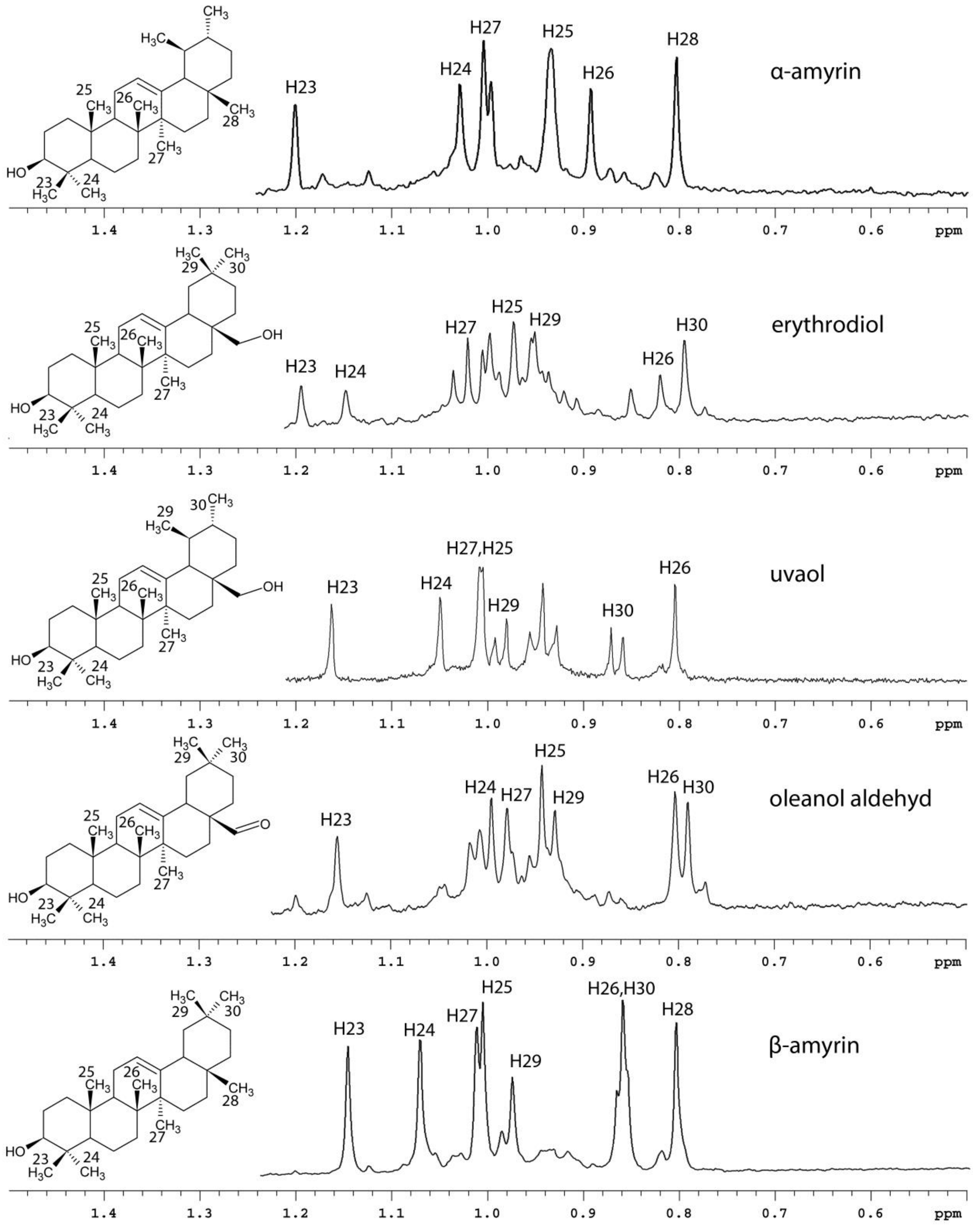

Figure S2. ^{1}H NMR spectra of identified phytosterols.

GC-MS and HR-MS

Agilent 6890 gas chromatograph coupled to Agilent 5973 mass spectrometer operating in 70 eV ionization mode (DB-5MS column (30 m × 0.25 mm × 0.25 mm), He as a carrier gas) was used for the verification of the collected phytosterol samples. Identification was based on the comparison of mass spectra with the MS spectra library.
High resolution of electrospray mass spectrum was measured using LTQ Orbitrap XL (Thermo Fisher Scientific). LTQ Orbitrap XL is a hybrid FT mass spectrometer combining a linear ion trap MS and the Orbitrap mass analyzer. The sample of a sterol conjugate was dissolved in MeOH and directly injected using 80 % MeOH as a mobile phase with the flow rate of 100uL/min. The following conditions were optimized for suitable ionization in the ESI source : sheat and auxiliary gas flow rate 35 and 10 a.u. of nitrogen, respectively, source voltage 4.3kV, capillary voltage 40 V, capillary temperature 275 °C, tube lens voltage 155V.

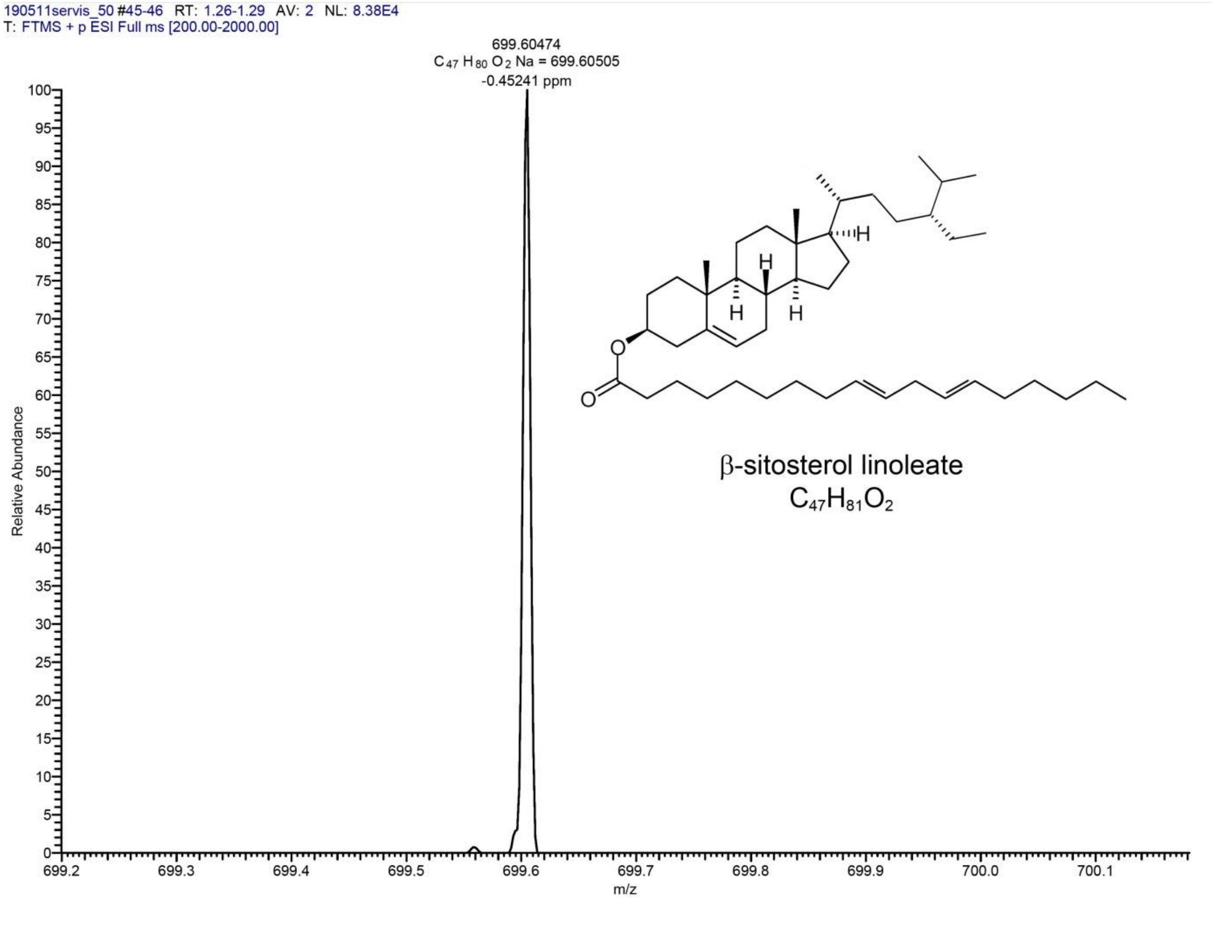

Figure S3. HR-MS spectrum of the isolated β-sitosterol linoleate.

Supercritical fluid extraction

The SFE experiments were carried out using a 150 mL extraction column (30 mm i.d.) filled with 30 g of dry matter (milled and sieved to particle fraction lower than 0.6 mm size), placed between layers of glass beads serving as solvent flow distributors. The extractor was immersed in a temperature-controlled water bath. Carbon dioxide (> 99.9 %, Linde Gas) was pressurised by a compressor (NovaSwiss 560.0007), controlled in a pressure regulator unit (NovaSwiss 560.0009) to operating pressure and pumped to the extractor. The solution leaving the extractor at its bottom was depressurised across a heated micrometer valve to atmospheric pressure and the extract was collected in pre-weighed glass traps. The amount of gaseous solvent leaving the trap was measured using a gas meter.

The experiment was performed at a temperature of 40 °C and a pressure of 280 bar. Carbon dioxide flow rate was adjusted to 0.8 g/min. The carbon dioxide-to-feed ratio, i.e. the mass of the solvent passed through the extractor per unit mass of the dry matter, was 18 g/g.

References

[S1] Li, T. S.; Beveridge, T. J.; Drover, J. C. Phytosterol content of sea buckthorn (Hippophae rhamnoides L.) seed oil: Extraction and identification. *Food Chem.* **2007**, *101*, 1633–1639.

[S2] Smallcombe S. H.; Patt S. L.; Keifer P. A. WET Solvent Suppression and Its Applications to LC NMR and High-Resolution NMR Spectroscopy. *J. Magn. Reson.* **1995**, *117*, 295-303.
